# Supplementary material for: Combination of the parent analogue of remdesivir (GS-441524) and molnupiravir results in a markedly potent antiviral effect in SARS-CoV-2 infected Syrian hamsters
Source: Front Pharmacol. 2022 Dec 20;13:1072202. doi: 10.3389/fphar.2022.1072202 (PMC9807602; doi:10.3389/fphar.2022.1072202)
Supplement: Supplementary file 1 [file Table1.DOCX]

**Supplementary Table 1: Detailed lung histopathology scoring**

| **Treatment group** | **hamster ID** | congestion | intralveolar hemorrhage | apoptotic bodies in bronchus wall | necrotizing bronchiolitis | Perivasc-ular edema | Bronchopneu-monia | % involved | perivascular inflammation | (endoth-elialitis) | (perivascular cuff) | peribronchial inflammation | Vascul-itis | **Cumulative Score** |
| --- | --- | --- | --- | --- | --- | --- | --- | --- | --- | --- | --- | --- | --- | --- |
| **Vehicle** | 1 | 1 |  | 1 |  |  | 0.5 | <5 | 1 | 1 | 1 | 1 |  | 4.5 |
|  | 2 | 1 |  | 1 |  |  | 0.5 | <5 | 0.5 | 0.5 | 0.5 | 0.5 |  | 3.5 |
|  | 3 | 1 |  | 1 |  |  | 0.5 | <5 | 1 | 1 | 1 | 0.5 |  | 4 |
|  | 4 | 1 |  | 1 |  |  | 1 | 20 | 1 | 1 | 1 | 0.5 |  | 5.5 |
|  | 5 | 1 |  | 1 |  |  | 1 | 20 | 1 | 1 | 1 | 0.5 |  | 4.5 |
|  | 6 | 1 |  | 1 |  |  | 0.5 | <5 | 0.5 | 0.5 | 0.5 | 1 | 0.5 | 4 |
|  | 7 | 1 |  | 1 |  |  | 0.5 | <5 | 1 | 1 | 1 | 1 |  | 4.5 |
|  | 8 | 1 |  | 1 |  |  | 1 | 10 | 1 | 1 | 1 | 1 |  | 5 |
|  | 9 | 1 |  | 1 |  |  | 0.5 | <5 | 0.5 | 0.5 | 0.5 | 0.5 |  | 3.5 |
|  | 10 | 1 |  | 1 |  |  | 0.5 | <5 | 1 | 1 | 1 | 1 |  | 4.5 |
|  | 11 | 1 |  | 1 |  |  | 0.5 | <5 | 1 | 1 | 1 | 1 |  | 4.5 |
|  | 12 | 1 |  | 1 |  |  |  |  | 0.5 | 0.5 | 0.5 | 1 |  | 3.5 |
| **EIDD-2801, 150 mg/kg BID** | 13 | 1 |  | 1 |  |  | 1 | 30 | 2 | 2 | 2 | 0.5 |  | 6.5 |
|  | 14 | 1 |  | 1 |  |  | 1 | 30 | 2 | 2 | 2 | 0.5 |  | 5.5 |
|  | 15 | 1 |  | 1 |  |  | 1 | 10 | 1 | 1 | 1 | 0.5 |  | 4.5 |
|  | 16 | 1 |  |  |  |  |  |  |  |  |  |  |  | 1 |
|  | 17 | 1 |  | 1 |  |  |  |  | 0.5 |  | 0.5 | 0.5 |  | 3 |
|  | 18 | 1 |  | 1 |  |  |  |  | 1 | 1 | 1 | 0.5 |  | 3.5 |
|  | 19 | 1 |  | 1 |  |  | 1 | 10 | 0.5 | 0.5 | 0.5 | 0.5 |  | 4 |
|  | 20 | 1 |  | 1 |  |  |  |  | 0.5 |  | 0.5 | 0.5 |  | 3 |
|  | 21 | 1 |  | 1 |  |  | 0.5 | <5 | 0.5 | 0.5 | 0.5 | 0.5 |  | 3.5 |
|  | 22 | 1 |  | 1 |  |  |  |  | 0.5 |  | 0.5 | 0.5 |  | 3 |
|  | 23 | 1 |  | 1 |  |  |  |  | 1 | 1 | 1 | 1 |  | 4 |
|  | 24 | 1 |  | 1 |  |  | 0.5 | <5 | 1 | 1 | 1 | 1 |  | 4.5 |
| **GS-441524, 50 mg/kg BID** | 25 | 1 |  |  |  |  |  |  | 0.5 |  | 0.5 |  |  | 1.5 |
|  | 26 | 1 |  |  |  |  |  |  | 0.5 |  | 0.5 |  |  | 1.5 |
|  | 27 | 1 | 1 | 1 |  |  |  |  | 0.5 | 0.5 | 0.5 | 0.5 |  | 4 |
|  | 28 | 1 | 1 | 1 |  |  |  |  | 0.5 | 0.5 | 0.5 | 0.5 |  | 4 |
|  | 29 | 1 |  | 1 |  |  |  |  | 0.5 | 0.5 | 0.5 | 0.5 |  | 3 |
|  | 30 | 1 | 1 |  |  |  |  |  | 0.5 |  | 0.5 |  |  | 2.5 |
|  | 31 | 1 | 1 |  |  |  |  |  | 0.5 | 0.5 | 0.5 | 0.5 |  | 3 |
|  | 32 | 1 |  | 1 |  |  |  |  | 1 | 1 | 1 | 0.5 | 0.5 | 4 |
|  | 33 | 1 |  | 1 |  |  |  |  | 0.5 |  | 0.5 | 0.5 |  | 3 |
|  | 34 | 1 |  | 1 |  |  | 1 | 20 | 2 | 2 | 2 | 0.5 |  | 5.5 |
|  | 35 | 1 |  | 1 |  |  |  |  | 0.5 |  | 0.5 | 0.5 |  | 3 |
|  | 36 | 1 |  | 1 |  |  |  |  | 0.5 |  | 0.5 | 0.5 |  | 3 |
| **combi EIDD+GS (150+50 mg/kg BID)** | 37 | 1 | 1 | 1 |  |  | 0.5 | <5 | 0.5 |  | 0.5 | 0.5 |  | 4.5 |
|  | 38 | 1 |  | 1 |  |  |  |  | 1 |  | 1 | 0.5 |  | 3.5 |
|  | 39 | 1 |  |  |  |  |  |  | 0.5 |  | 0.5 |  |  | 1.5 |
|  | 40 | 1 |  | 1 |  |  |  |  | 0.5 |  | 0.5 | 0.5 |  | 3 |
|  | 41 | 1 |  |  |  |  |  |  | 0.5 |  | 0.5 | 0.5 |  | 2 |
|  | 42 | 1 |  |  |  |  |  |  | 0.5 | 0.5 | 0.5 | 0.5 |  | 2 |
|  | 43 | 1 |  |  |  |  |  |  | 0.5 |  | 0.5 |  |  | 1.5 |
|  | 44 | 1 |  | 1 |  |  |  |  | 0.5 | 0.5 | 0.5 | 0.5 |  | 3 |
|  | 45 | 1 |  |  |  |  |  |  | 0.5 |  | 0.5 |  |  | 1.5 |
|  | 46 | 1 |  | 1 |  |  |  |  | 0.5 |  | 0.5 | 0.5 |  | 3 |

**Parameters between () are not included in the cumulative score*
