# Supplementary material for: Combination of the parent analogue of remdesivir (GS-441524) and molnupiravir results in a markedly potent antiviral effect in SARS-CoV-2 infected Syrian hamsters
Source: Front Pharmacol. 2022 Dec 20;13:1072202. doi: 10.3389/fphar.2022.1072202 (PMC9807602; doi:10.3389/fphar.2022.1072202)
Supplement: Supplementary file 2 [file Image1.pdf]

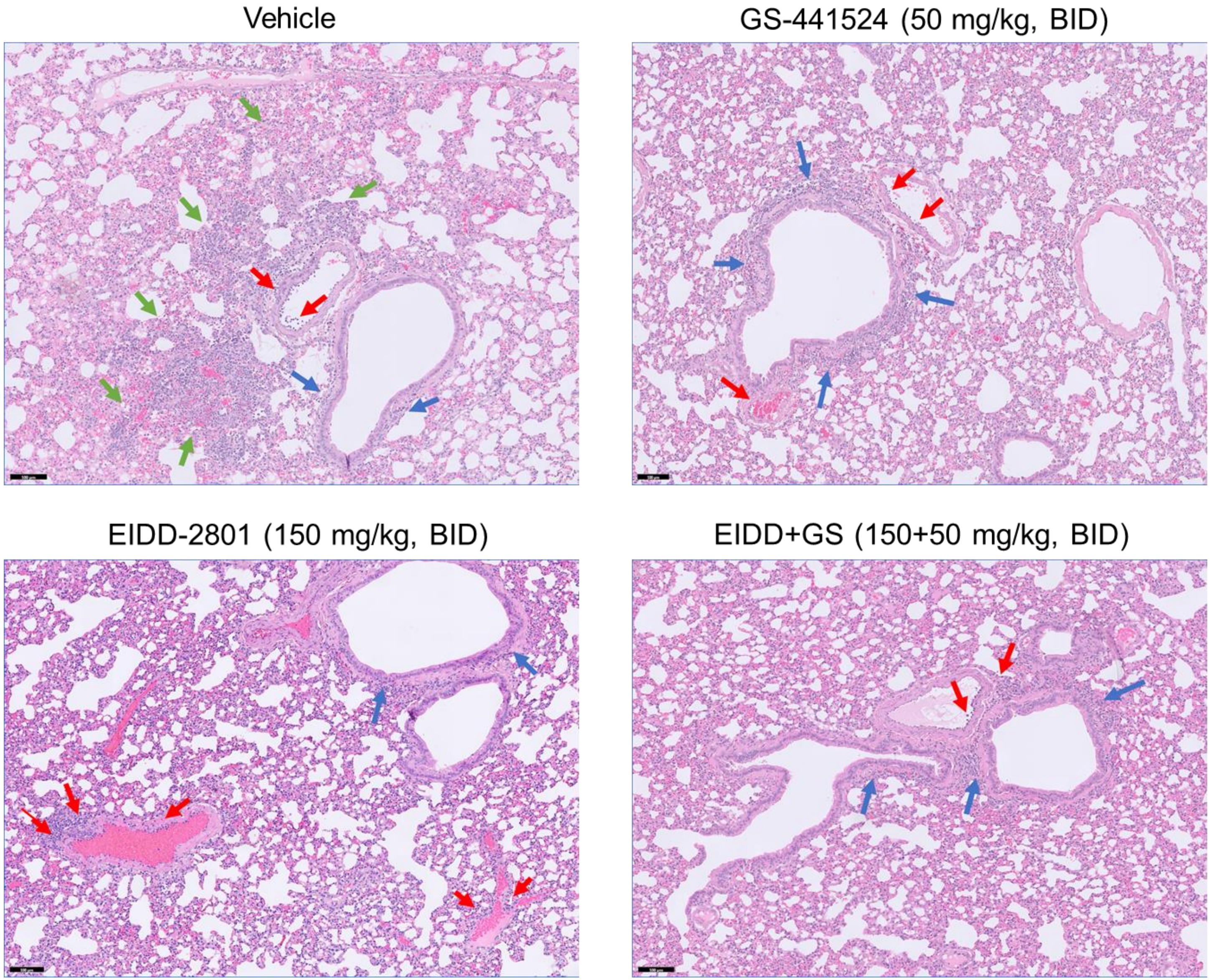

Fig. S1. Histopathology of lungs of Syrian hamsters treated with Molnupiravir (EIDD-2801), GS-441524 or a combination of both compounds. Representative H&E images of lungs of control (vehicle-treated), EIDD-2801-treated (150 mg/kg, BID), GS-441524-treated (50 mg/kg, BID) and combination-treated (EIDD-2801+GS-441524 at 150+50 mg/kg, BID, respectively) SARS-CoV-2–infected hamsters at day 4 post-infection (pi). The lungs of vehicle-treated infected hamsters show several regions with bronchopneumonia (green arrows), peri-vascular inflammation with endotheliatitis (red arrows) and limited peri-bronchial inflammation (blue arrows), whereas the lungs of compounds treated hamsters show improved lung pathology. Less signs of pathology are detected in the lungs from the combination-treated group. Scale bars, 100 µm.
